# Stability SCAD: a powerful approach to detect interactions in large-scale genomic study

**DOI:** 10.1186/1471-2105-15-62

**Published:** 2014-03-01

**Authors:** Jianwei Gou, Yang Zhao, Yongyue Wei, Chen Wu, Ruyang Zhang, Yongyong Qiu, Ping Zeng, Wen Tan, Dianke Yu, Tangchun Wu, Zhibin Hu, Dongxin Lin, Hongbing Shen, Feng Chen

**Affiliations:** 1 Department of Epidemiology and Biostatistics and Ministry of Education (MOE) Key Lab for Modern Toxicology, School of Public Health, Nanjing Medical University, Nanjing, China; 2 State Key Laboratory of Molecular Oncology and Department of Etiology and Carcinogenesis, Cancer Institute and Hospital, Chinese Academy of Medical Sciences and Peking Union Medical College, Beijing, China; 3 Section of Clinical Epidemiology, Jiangsu Key Laboratory of Cancer Biomarkers, Prevention and Treatment, Cancer Center, Nanjing Medical University, Nanjing, China; 4 Institute of Occupational Medicine and Ministry of Education Key Laboratory for Environment and Health, School of Public Health, Tongji Medical College, Huazhong University of Science and Technology, Wuhan, China; 5 State Key Laboratory of Reproductive Medicine, Nanjing Medical University, Nanjing, China; 6 Department of Mathematical and Statistical Sciences, Nanjing Forestry University, Nanjing, China

**Keywords:** Genome-wide association study (GWAS), Interaction, Least absolute shrinkage and selection operator (LASSO), Penalized logistic regression, Smoothly clipped absolute deviation (SCAD), Stability selection

## Abstract

**Background:**

Evidence suggests that common complex diseases may be partially due to SNP-SNP interactions, but such detection is yet to be fully established in a high-dimensional small-sample (small-n-large-p) study. A number of penalized regression techniques are gaining popularity within the statistical community, and are now being applied to detect interactions. These techniques tend to be over-fitting, and are prone to false positives. The recently developed stability least absolute shrinkage and selection operator (_S_LASSO) has been used to control family-wise error rate, but often at the expense of power (and thus false negative results).

**Results:**

Here, we propose an alternative stability selection procedure known as stability smoothly clipped absolute deviation (_S_SCAD). Briefly, this method applies a smoothly clipped absolute deviation (SCAD) algorithm to multiple sub-samples, and then identifies cluster ensemble of interactions across the sub-samples. The proposed method was compared with _S_LASSO and two kinds of traditional penalized methods by intensive simulation. The simulation revealed higher power and lower false discovery rate (*FDR*) with _S_SCAD. An analysis using the new method on the previously published GWAS of lung cancer confirmed all significant interactions identified with _S_LASSO, and identified two additional interactions not reported with _S_LASSO analysis.

**Conclusions:**

Based on the results obtained in this study, _S_SCAD presents to be a powerful procedure for the detection of SNP-SNP interactions in large-scale genomic data.

## Background

High-dimensional genomic data are becoming increasingly available to assist in the identification of genetic factors for complex diseases such as lung cancer. In particular, genome-wide association studies (GWAS) have implicated a variety of genetic variants in many diseases, while only a small fraction of phenotypic variation was explained by those. This suggests that multi-locus gene-gene or gene-environment interactions must be considered [1].

Gene-gene interactions could be detected using a variety of methods [2]. For example, multifactor dimensionality reduction (MDR, [3]) is a non-parametric and model-free method that does not require any assumption of genetic mode of inheritance. However, MDR is inefficient in handling large scale genetic datasets [4]. Penalized regression methods such as least absolute shrinkage and selection operator (LASSO) [5] and smoothly clipped absolute deviation (SCAD) [6] are also widely used for high-dimensional data. LASSO is a useful tool for detecting gene-gene interactions with a broad range of simulations [7]. SCAD penalty has an oracle property, and thus it is more consistent with the actual effects than LASSO [6]. The cross-validation is usually used for the choice of the amount of regularization in penalized regression methods (e.g., LASSO and SCAD), but it often includes too many noise variables. In an attempt to minimize such a problem, a modified LASSO penalized method, stability LASSO (_S_LASSO), has been proposed to unify optimal shrinkage and variable selection in GWAS ([8]). Stability selection controls false discovery rate and renders cross-validation practically unnecessary. Alexander and Lange (2011) claimed that _S_LASSO could accurately identify the most important regions of GWAS, but in a simulation study _S_LASSO offers less power than the simpler and less computationally intensive methods of marginal association testing [8].

It has been shown that the LASSO penalty could produce a bias even in the simple regression setting due to its linear increase of penalty on regression coefficients. To remedy this bias issue, a non-concave penalty such as SCAD penalty was proposed. SCAD has the so-called oracle property, meaning that, in the asymptotic sense, it performs as effectively as if an analyst had known in advance which coefficients were zero and which ones were nonzero [6]. SCAD is capable of achieving the sparse estimator in combination with smaller biases in linear regression than LASSO. Here, we propose a new stability selection procedure in combination with SCAD penalization (_S_SCAD). The new method was compared to _S_LASSO using systematic simulations and a published GWAS study.

## Methods

### Ethics statement

This collaborative study was approved by the institutional review boards of China Medical University, Tongji Medical College, Fudan University, Nanjing Medical University, and Guangzhou Medical College with written informed consent from all participants.

### Penalized logistic regression for case-control GWAS

Let *y*_*i*_ denote the disease status of the individual *i* ( *i* =1,…,*n*): 1 for case and 0 for control. The SNP of individual *i*, *x*_*ij*_, is formatted as the count of a particular allele (0, 1, or 2) where *j* = 1,…,*m*. The logistic model below includes SNP-SNP interaction terms:

(1) $$\begin{array}{c} y_{i}∼\mathrm{Binominal}\left(1,\pi_{i}\right),\\ \log\frac{\pi_{i}}{1-\pi_{i}}=\beta_{0}+\sum_{j=1}^{m}\beta_{j}x_{\mathrm{ij}}+\sum_{j<k}\xi_{\mathrm{jk}}x_{\mathrm{ij}}x_{\mathrm{ik}},i=1,\cdots n, \end{array}$$

where *x*_*ij*_ and *x*_*ij*_*x*_*ik*_ are main effect and interaction features, respectively.

Penalized likelihood method makes the fitting of a logistic model with small-n-large-p computationally feasible. It also provides a mechanism for feature selection. *L(θ)* denotes the likelihood function of the above logistic model (1), where *θ* consists of those *β* and *ξ*. The penalized log-likelihood function takes the form

(2) $$l_{p}\left(\theta \right)=-2\log L\left(\theta \right)+\sum_{j}p_{\lambda }\left(\theta_{j}\right),$$

where *p*_*λ*_(•) is the penalty function characterized by a tuning parameter λ. The following penalty functions are used in LASSO and SCAD, respectively:

$$\begin{array}{l} \mathrm{LASSO}\;\mathrm{penalty}:\;p_{\lambda }\left(\theta_{j}\right)=\lambda |\theta_{j}|,\\ \mathrm{SCAD}\;\mathrm{penalty}:\;p_{\lambda }^{'}\left(\left|\theta \right|\right)=\lambda \left\{1\left(|\theta |\leq \lambda \right)+\frac{{\left(\mathrm{a\lambda}-|\theta |\right)}_{+}}{\left(a-1\right)\lambda }1\left(|\theta |>\lambda \right)\right\}, \end{array}$$

where *a* is a fixed constant larger than 2, the notation (**·**)_+_ stands for the positive part, and 1(**·**) denotes the indicator function.

When the penalized logistic regression model is fitted, a predetermined number of the components of *θ* can be forced to zero by tuning *λ* to a certain value. For a specific variable, estimation of the coefficient is non-zero if the coefficient exceeds the threshold or equals to zero. Thus the selection of tuning parameter is a crucial step at the application of penalized likelihood. This is usually accomplished with cross validation. We used cross validation predictive area under the *ROC* curve to choose the appropriate tuning parameter.

LASSO and SCAD with cross-validated tuning parameter selection often lead to the inclusion of too many noise variables for high-dimensional data [9]. For variable selection in small-n-large-p genomic data, choosing the amount of regularization is more challenging than predicting where a cross-validation scheme can be used. A false variable in variable selection may lead to apparent association with a disease phenotype either through chance or correlation with the true variables. Studies using high-dimensional data often need to be validated due to false variables. Another practical issue here is reducing false variables while maintaining the power to detect relevant variables. To address this problem, Meinshausen and Bülmann [9] proposed a stability selection procedure that is relatively insensitive to the choice of tuning parameter [9].

In the current study, SCAD was used in variable selection in each sub-sample, and then stability selection was used to identify consensus ensemble of solutions.

### Stability selection procedure

a) Meinshausen and Bülmann (MB) stability selection methodology

Stability selection seeks to identify the non-zero entries *S =* {*k:θ*_*k*_ *≠* 0} of a sparse parameter vector in above penalized logistic regression model (2). Assuming that the set *I* is a uniform random sub-sample of the index set {*1,…,n*}, the index set was used to subsample from the data to yield a subset *Z(I)*. For the subset and a given regularization parameter *λ* ∈ Λ, penalized regression procedure was used to yield an estimate of *θ*_*k*_, i.e., ${\hat{\theta }}_{k}^{\lambda }\left(I\right)$. Selection variable set was denoted as

$${\hat{S}}^{\lambda }\left(I\right)=\left\{k:{\hat{\theta }}_{k}^{\lambda }\neq 0\right\}.$$

The conditional selection probability of the *k*-th covariate was defined as

(3) $${\hat{\Pi }}_{k}^{\lambda }=P\left\{k\in {\hat{S}}^{\lambda }\left(I\right)|X,y\right\}.$$

The selection probabilities were naturally estimated by Monte Carlo method averaging over *B* times independent sub-sampling. Variables with high selection probabilities were retained, while those with low selection probabilities were discarded. For a cut-off *π*_*thr*_ with 0 < *π*_*thr*_ < 1 and a set of regularization parameters Λ, the stable selection variables set was defined as

(4) $${\hat{S}}^{\mathrm{stable}}=\left\{k:\max_{\lambda \in \Lambda }{\hat{\Pi }}_{k}^{\lambda }\geq \pi_{\mathrm{thr}}\right\}.$$

The basic idea of the stability selection is to repeat the feature selection process in many randomly perturbed subsamples (e.g., by bootstrapping the samples in the original data set), and to include features that are relevant to majority of the subsamples. The baseline of the stability selection procedure is explained below:

Given a cut-off, compute the stable selection variables set ${\hat{S}}^{\mathrm{stable}}=\left\{k:\max_{\lambda \in \Lambda }{\hat{\Pi }}_{k}^{\lambda }\geq \pi_{\mathrm{thr}}\right\}$.

The threshold value π_*thr*_ is a tuning parameter whose influence is very small. In principle, the tuning parameter of MB is based on the following theorem 1 of Meinshausen and Bülmann.

***Theorem 1 (error control).*** Assuming that the distribution of $\left\{1_{\left\{k\in {\hat{S}}^{\lambda }\right\}},k\in N\right\}$ is exchangeable for *λ* ∈ Λ, and the original procedure is not worse than random guessing. Let *q*_*Λ*_ be the average number of selected variables, $q_{\Lambda }=E|\underset{\lambda \in \Lambda }{\cup }{\hat{S}}^{\lambda }\left(I\right)|$, the expected number V of falsely selected variables is then bounded for *π*_*thr*_ ∈ (1/2, 1] by

(5) $$E\left(V\right)\leq \frac{1}{2\pi_{\mathrm{thr}}-1}\frac{q_{\Lambda }^{2}}{p}.$$

b) Improvements of the MB stability selection

In the current study (where *p* ≫ *n*), the primary goal was controlling the false discovery rate (*FDR*):

(6) $$\begin{array}{ll} \mathrm{FDR} & =E\left(V/\left|{\hat{S}}^{\mathrm{stable}}\right|\right)\approx E\left(V\right)/q_{\Lambda }\leq \frac{1}{2\pi_{\mathrm{thr}}‒1}\frac{q_{\Lambda }}{p}\\ & =\frac{1}{2\pi_{\mathrm{thr}}‒1}\frac{q_{\Lambda }}{m+m\left(m‒1\right)/2}. \end{array}$$

An advantage of the stability selection is that the choice of the regularization parameters Λ does not have strong influence on the results, as long as λ is varied within a reasonable range [9]. To control *FDR*, we first chose a fixed regularization region Λ, and then chose the selective probability threshold π_*thr*_ according to the above inequality (6).

We set a fixed regularization region as Λ = [λ_min_, λ_max_], which was decided by the number of selected variables ***q*** as follow: λ_max_ corresponded to the variable that first entered the regularization path and λ_min_ was chosen such that the first ***q*** variables that appeared in the regularization path, mathematically, λ_min_ was chosen such that $\left|\cup_{\lambda_{\text{min}}\leq \lambda \leq \lambda_{\text{max}}}{\hat{S}}^{\lambda }\right|\leq q$. The value of ***q*** was chosen a priori to yield a non-trivial bound (see discussion on the paper by Meinshausen and Bühlmann [9]), i.e. $q=O\left(\sqrt{\left(2\pi_{\mathrm{thr}}-1\right)p}\right)$). The choice of ***q*** in stability selection does not have a strong impact on the *FDR*[9]. We used a conservative estimate of ***q*** (the square root of the number of predictors) in the discovery stage.

For the fixed regularization region, we applied the SCAD procedure to every subsample. $q_{\Lambda }=E|\underset{\lambda \in \Lambda }{\cup }{\hat{S}}^{\lambda }\left(I\right)|$ was estimated via the Monte Carlo simulation averaging over *B* times independent sub-sampling. The threshold value *π*_*thr*_ was solved while maintaining *FDR ≤ α* according to the expression (6) as

(7) $$\pi_{\mathrm{thr}}=\left(1+\frac{q_{\Lambda }}{\mathrm{p\alpha}}\right)/2,\;\mathrm{if}\;q_{\Lambda }\leq \mathrm{p\alpha}.$$

Unfortunately, given the nature of genetic data, the exact hypotheses required by the theorem of Meinshausen and Bülmann are unlikely to hold [9]. In particular, the exchangeability assumption of Theorem 1 about the indicator random variables $\left\{1_{\left\{k\in {\hat{S}}^{\lambda }\right\}},k\in N\right\}$ is questionable due to the correlations among the markers induced by linkage disequilibrium. We worried that the false positives of stability selection might be grossly wrong in our genetic data. So we adopted the method described in Alexander and Lange [8] to make a rough check on the false discovery rate of stability selection. We randomly permuted the phenotype vector *y* for all participants, firstly. We then performed the stability selection procedure on the permutation sample and obtained the selection probability of the variable corresponding to the maximum test statistic in the association analysis, and finally compared the selection probability with the cut-off calculated from the theorem of Meinshausen and Bülmann.

### Data simulation

_S_SCAD selection procedure was compared with LASSO, SCAD, and _S_LASSO under a variety of interaction models.

#### Genotype simulation

**Figure 1:**
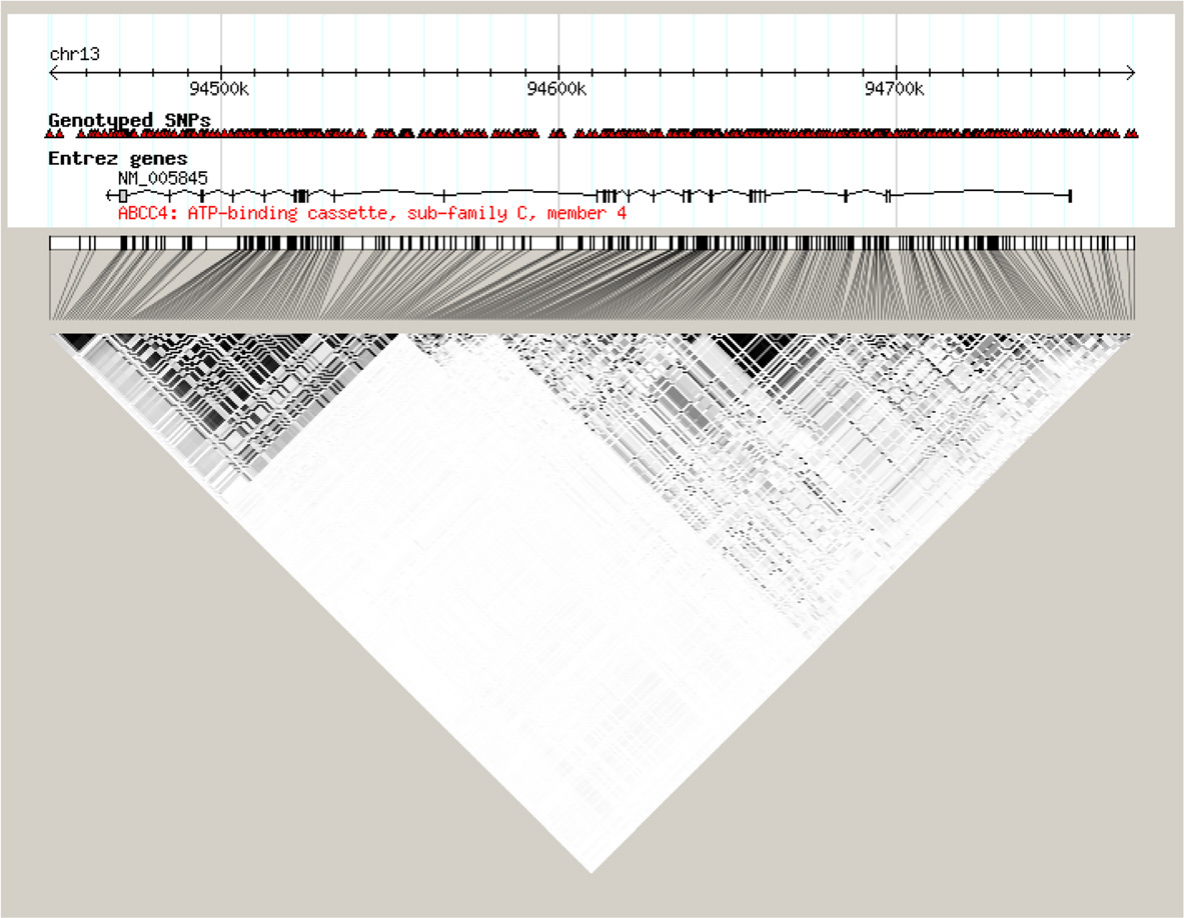
**Pairwise r**^**2**^** among the 327 SNPs across the gene *ABCC4*.** The color of each box signifies the value of r^2^ between SNPs alleles, with the black indicating strongest relationship between a pair of marks (1 = black, 0 = white).

HAPGEN (v2.2.0) program [10] was used to simulate genotype information. The simulation parameters for SNP frequencies and variance structure were extracted from HapMap3 JPT + CHB that includes SNPs located within ±20-kb of *ABCC4* (ATP-binding cassette sub-family C member 4) at 13q31. The legend file for the SNP markers, and the fine scale recombination rate were downloaded from the HapMap website (http://hapmap.ncbi.nlm.nih.gov/downloads/index.html.en). After quality control, 327 common SNPs remained (with the exclusion criteria: missing data of SNPs > 5%, minor allele frequence < 5%, Hardy-Weinberg *p*-value < 10^-4^). The linkage disequilibrium (LD) pattern is shown in Figure 1.

#### Phenotype simulations

Genetic interaction model was applied for case/control phenotypes simulations. During the phenotype simulation, we took *m* =327 ($C_{m}^{2}=53301$ two-way interactions) and randomly selected causal SNPs from different haplotype blocks. The blocks were computed through Haploview v4.2 by standard expectation-maximization algorithm [11], which partitioned the region into segments of strong linkage disequilibrium (LD). A total of 26 blocks were generated with a minimum size of 2 markers and a maximum size of 64 markers. All the causal SNPs and SNP-SNP interactions were assumed to improve the risk (OR = 1.3, 1.4, and 1.5, respectively); wherein we let y_*i*_ denote the phenotype value of subject *i*, which is obtained according to the logistic regression model (1).

We conducted simulations to evaluate selection performance of the LASSO, SCAD, _S_LASSO and _S_SCAD procedures under the following scenarios:

(A). The interactive SNPs have no main effects:

*β*_*j*_ = 0 for all *j*, and *ξ*_*jk*_ ≠ 0 for some randomly chosen *j, k.*

(B). Only one SNP in the SNP-SNP interaction pair has a main effect:

*ξ*_*jk*_ ≠ 0 and *β*_*j*_ ≠ 0 for some randomly chosen *j, k.*

(C). Both interactive SNPs have main effects:

*ξ*_*jk*_ ≠ 0, *β*_*j*_ ≠ 0 and *β*_*k*_ ≠ 0 for some randomly chosen *j, k.*

The odds ratio parameters are shown in Table 1. Since there are only a few etiological loci - only a few of the coefficients in the model are nonzero - the phenomenon is referred to as being sparse.

**Table 1:** Parameter settings of the different kinds of scenarios

**Feature of simulated set**	**Scenario**	**Number of nonzero main effects**	**Number of nonzero interactions**	**Locations of causal variants**	**Designed OR**
*ABCC4* (327 SNPs, 53301 two-way interactions)	A1/A2/A3	0	1	SNP33 × SNP197	1.5/1.4/1.3
	B1/B2/B3	1	1	SNP18 + SNP18 × SNP134	1.5/1.4/1.3
	C1/C2/C3	2	1	SNP33 + SNP134 + SNP33 × SNP134	1.5/1.4/1.3

*Abbreviations*: *ABCC4* ATP-binding cassette sub-family C member 4, *OR* odds ratio, *SNP* single nucleotide polymorphisms.

For every simulation scenario of phenotype in Table 1, the phenotype *y*_*i*_ was generated based on the simulated SNPs by HAPGEN 2 using the above-mentioned logistic regression model (1). We simulated the population with an equal number of cases and controls (n/2 = 10,000) with 200 replicate data sets, and then 1,000 cases and 1,000 controls were randomly sampled from the population to form one sample set. Next, we performed different variable selection methods for each sample set.

#### Simulated data analysis

R software (version 2.14.0, The R Foundation for Statistical Computing) was used to perform the simulation. The package “glmnet” and modified package “ncvreg” were used for LASSO and SCAD analysis, respectively. For stability selection, we chose *B* = 500 times independent sub-samples with a size of 500 cases and 500 controls from each 1000-1000 cases-controls sample set.

### Application

The study subjects were from an ongoing two-center (Nanjing and Beijing, China) GWAS of lung cancer in China. At recruitment, written informed consent was obtained from each subject. The study was approved by the institutional review boards of each participating institution. The details of population and other related information were described previously [12]. A systematic quality control procedure was applied for both SNPs and individuals. SNPs were excluded if they did not map on autosomal chromosomes, with minor allele frequency < 0.05, with call rate < 95%, with *p* < 1 × 10^-5^ for Hardy-Weinberg equilibrium in combined samples of two studies or *p* < 1 × 10^-4^ in either the Nanjing or Beijing study samples. We removed samples with a call rate of < 95%, ambiguous gender, familial relationships, extreme heterozygosity rate, and outliers. Briefly, there were 1,473 cases and 1,962 controls in the Nanjing center, 858 cases and 1,115 controls in the Beijing center after quality control.

A multi-stage strategy is often used for detecting interactions on a genome-wide scale. The method proposed in the current study could not be directly applied to genome-wide scale SNPs data since it is too computationally intensive to exhaustively search for all SNP pairs. A filtering method could be helpful, as explained below using the achPathway pathway (a role of nicotinic acetylcholine receptors in the regulation of apoptosis). This pathway is one of the top pathways associated with lung cancer risk in the Han Chinese population. Several studies have shown that the nicotinic acetylcholine receptors can induce cell proliferation as well as angiogenesis [13]. The achPathway pathway includes the genes *PIK3R1, PTK2B, PTK2, AKT1, PIK3CG, FASLG, MUSK, CHRNG, RAPSN, BAD, FOXO3, TERT, CHRNB1, PIK3CA, SRC* and *YWHAH*. All SNPs are mapped into genes within 20 kb downstream or upstream. All together, there are 344 common SNPs. We conducted an exhaustive search ($C_{344}^{2}=58996$) of two-way interaction in the pathway. Covariates including age, gender, pack-year of smoking, and the first two principal components, which have been proposed to sufficiently adjust for population stratification derived from EIGENSTRAT 3.0 [14], were adjusted in the stability selection procedure [12].

To increase confidence in the selection of significant interactions from tens of thousands of SNP pairs, interactions findings often need to be replicated in independent studies. We adopted a two-stage strategy in the current study. In the initial discovery stage, we used _S_LASSO and _S_SCAD to select significant SNP-SNP interactions using the data from the Nanjing center. In the replication stage, the findings in the initial step were validated using the data from the Beijing center with _S_LASSO and _S_SCAD. A slight variation was made to calibrate the significant threshold for the replication phase (i.e., we set the initial fixed number of variables in Beijing study as the number of selected variables in the discovery stage).

The SNP pairs were selected using the following criteria: (i) the interaction had the selection probability *π*_*l*_ ≥ *π*_*thr*1_ in the Nanjing study, while in the Beijing study the selection probability was *π*_*l*_ ≥ *π*_*thr*2_ (*π*_*thr*1_ and *π*_*thr*2_ are the significant thresholds of the Nanjing and Beijing studies, corresponding to the control of the *FDR* under 0.1); (ii) the Nanjing and Beijing centers both demonstrated identical direction of odds ratios for the two SNPs, with their interaction derived from penalized logistic regression.

## Results

### Result of simulation

We evaluated the performance of different variable selection procedures using four established statistical indexes, including the true positive rate (*TPR*):

$$\mathrm{TPR}=\frac{\mathrm{TP}}{\mathrm{TP}+\mathrm{FN}},$$

the Mathhews correlation coefficient (*MCC*) [15]:

$$\mathrm{MCC}=\frac{\mathrm{TP}\times \mathrm{TN}-\mathrm{FP}\times \mathrm{FN}}{\sqrt{\left(\mathrm{TP}+\mathrm{FP}\right)\left(\mathrm{TP}+\mathrm{FN}\right)\left(\mathrm{TN}+\mathrm{FP}\right)\left(\mathrm{TN}+\mathrm{FN}\right)}},$$

the estimated area under the *ROC* curves (*AUC*) [16]:

$$\mathrm{AUC}=\frac{1}{t_{0}\left(p-t_{0}\right)}\sum_{u=1}^{t_{0}}\sum_{v=t_{0}+1}^{p}\left[I\left({\hat{p}}_{u}>{\hat{p}}_{v}\right)+\frac{1}{2}I\left({\hat{p}}_{u}={\hat{p}}_{v}\right)\right],$$

and the estimated false discovery rate (*FDR,*[17]):

$$\mathrm{FDR}=\frac{\mathrm{FP}}{\mathrm{FP}+\mathrm{TP}},$$

where *TP* and *TN* stand for true positives and true negatives, *FP* and *FN* stand for false positives and false negatives, respectively. ${\hat{p}}_{u}$ is the selection probability of the *u*-th predictor and the first *t*_0_ variables are assumed to be true signals. The index *TPR* is known as sensitivity, whereas *MCC* is generally regarded as a balanced measure for both sensitivity and specificity, *AUC* summarizes overall prediction performance, and *FDR* is a criterion to measure and control the number of false positives for the class-skewed high-throughput data [18]. The indexes *TPR, MCC* and *AUC* are used to measure the power of detecting interactions, while *FDR* is primarily used to assess false positives of detection.

The simulation results of different procedures for the three kinds of scenarios are summarized in Tables 2, 3 and 4. All indexes are presented as average and standard error using 200 replications. The simulation results based on the Tables 2, 3 and 4 are described from the following two perspectives.

**Table 2:** Selection performance of different methods in different scenarios (OR = 1.5)

	**Method**	**Scenario**		
		**A1**	**B1**	**C1**
*TPR*	LASSO	0.768(0.027)	0.775(0.020)	0.857(0.018)
	SCAD	0.801(0.025)	0.775(0.021)	0.603(0.016)
	_S_LASSO	0.750(0.018)	0.780(0.009)	0.990(0.005)
	_S_SCAD	0.933(0.013)	0.897(0.002)	0.968(0.003)
*MCC*	LASSO	0.292(0.014)	0.384(0.012)	0.356(0.021)
	SCAD	0.350(0.013)	0.435(0.013)	0.373(0.016)
	_S_LASSO	0.776(0.011)	0.706(0.012)	0.875(0.011)
	_S_SCAD	0.826(0.008)	0.843(0.016)	0.837(0.015)
*AUC*	LASSO	0.593(0.018)	0.601(0.002)	0.632(0.002)
	SCAD	0.596(0.018)	0.606(0.002)	0.632(0.002)
	_S_LASSO	0.612(0.001)	0.616(0.002)	0.638(0.005)
	_S_SCAD	0.631(0.001)	0.615(0.001)	0.630(0.002)
*FDR*	LASSO	0.859(0.013)	0.827(0.007)	0.789(0.026)
	SCAD	0.837(0.009)	0.813(0.008)	0.742(0.018)
	_S_LASSO	0.186(0.006)	0.160(0.009)	0.107(0.017)
	_S_SCAD	0.167(0.003)	0.147(0.007)	0.166(0.014)

*Abbreviations*: *TPR* true positive rate, *MCC* Matthews correlation coefficient, *AUC* area under the *ROC* curve, *FDR* false discovery rate.

Numbers in each cell represent mean (standard error) by 200 times simulation.

**Table 3:** Selection performance of different methods in different scenarios (OR = 1.4)

	**Method**	**Scenario**		
		**A2**	**B2**	**C2**
*TPR*	LASSO	0.726(0.028)	0.726(0.021)	0.797(0.020)
	SCAD	0.750(0.026)	0.740(0.021)	0.734(0.022)
	_S_LASSO	0.706(0.019)	0.720(0.012)	0.942(0.010)
	_S_SCAD	0.861(0.016)	0.890(0.004)	0.953(0.002)
*MCC*	LASSO	0.240(0.015)	0.334(0.013)	0.309(0.013)
	SCAD	0.295(0.014)	0.380(0.015)	0.333(0.014)
	_S_LASSO	0.716(0.012)	0.636(0.015)	0.826(0.013)
	_S_SCAD	0.787(0.009)	0.800(0.018)	0.817(0.018)
*AUC*	LASSO	0.537(0.018)	0.542(0.002)	0.577(0.003)
	SCAD	0.538(0.019)	0.529(0.003)	0.570(0.004)
	_S_LASSO	0.557(0.002)	0.569(0.003)	0.590(0.003)
	_S_SCAD	0.557(0.003)	0.561(0.003)	0.560(0.001)
*FDR*	LASSO	0.868(0.014)	0.829(0.010)	0.797(0.008)
	SCAD	0.838(0.009)	0.817(0.010)	0.754(0.009)
	_S_LASSO	0.198(0.007)	0.146(0.009)	0.122(0.010)
	_S_SCAD	0.166(0.004)	0.149(0.007)	0.180(0.007)

*Abbreviations*: *TPR* true positive rate, *MCC* Matthews correlation coefficient, *AUC* area under the *ROC* curve, *FDR* false discovery rate.

Numbers in each cell represent mean (standard error) by 200 times simulation.

**Table 4:** Selection performance of different methods in different scenarios (OR = 1.3)

	**Method**	**Scenario**		
		**A3**	**B3**	**C3**
*TPR*	LASSO	0.681(0.032)	0.664(0.023)	0.777(0.021)
	SCAD	0.715(0.028)	0.678(0.023)	0.7853(0.022)
	_S_LASSO	0.651(0.019)	0.692(0.012)	0.874(0.012)
	_S_SCAD	0.835(0.014)	0.808(0.005)	0.878(0.005)
*MCC*	LASSO	0.173(0.018)	0.298(0.018)	0.303(0.016)
	SCAD	0.253(0.016)	0.350(0.017)	0.312(0.014)
	_S_LASSO	0.676(0.012)	0.600(0.015)	0.768(0.013)
	_S_SCAD	0.736(0.012)	0.737(0.014)	0.778(0.019)
*AUC*	LASSO	0.506(0.018)	0.524(0.003)	0.536(0.006)
	SCAD	0.517(0.021)	0.514(0.006)	0.536(0.006)
	_S_LASSO	0.518(0.002)	0.545(0.005)	0.535(0.005)
	_S_SCAD	0.537(0.005)	0.541(0.002)	0.551(0.002)
*FDR*	LASSO	0.869(0.015)	0.838(0.011)	0.798(0.010)
	SCAD	0.832(0.013)	0.819(0.008)	0.726(0.009)
	_S_LASSO	0.188(0.007)	0.164(0.006)	0.182(0.010)
	_S_SCAD	0.169(0.006)	0.163(0.007)	0.174(0.008)

*Abbreviations*: *TPR* true positive rate, *MCC* Matthews correlation coefficient, *AUC* area under the *ROC* curve, *FDR* false discovery rate.

Numbers in each cell represent mean (standard error) by 200 times simulation.

#### (I). _S_LASSO/_S_SCAD has lower false discovery rate than LASSO/SCAD while possessing similar AUC

It appears that _S_LASSO and _S_SCAD have lower $\overset{⌢}{\mathrm{FDR}}$ for identifying interactions in comparison to LASSO or SCAD. Contrary to LASSO and SCAD which generated unacceptably high $\overset{⌢}{\mathrm{FDR}}$ in all scenarios, both _S_LASSO and _S_SCAD controlled $\overset{⌢}{\mathrm{FDR}}$ at an acceptable level. In regards to predictive *AUC*, there was no difference in stability selection procedures that being its inclusion or exclusion. In other words, _S_LASSO or _S_SCAD achieved a higher specificity than other procedures despite the similar diagnostic accuracy of *AUC.*

#### (II). _S_SCAD has more robust power against _S_LASSO among different interaction models

Given an acceptable $\overset{⌢}{\mathrm{FDR}}$ level, we compared _S_SCAD with the _S_LASSO procedure in the detection of SNP-SNP interactions. _S_LASSO lost its ability to rapidly detect interactions as the reduction of the main effects from the scenarios C1/C2/C3 to scenarios B1/B2/B3 and A1/A2/A3. _S_SCAD, on the other hand, possessed robust detecting power under all scenarios. For the scenario A1/A2/A3, in which the model only included the SNP-SNP interaction without any main effects of SNPs, _S_SCAD was more powerful than _S_LASSO. An exemplification of this can be seen in scenario A1, in which the criteria of measuring the power of variable selection procedures echoed the trend: therein the *TPR* of _S_SCAD was 93.3%, while the one of _S_LASSO was only roughly 75.0%. Likewise, *MCC* and *AUC* were also both higher with _S_SCAD than with _S_LASSO.

The underlying interactions were better detected with _S_LASSO in the scenario C1 where the corresponding main effects were not too small (Table 2). _S_LASSO possessed slightly higher *TPR, MCC* and *AUC* than _S_SCAD in the scenario C1. _S_SCAD was more powerful than _S_LASSO in the scenario C2/C3 where the corresponding main effects ranged from small to moderate (Tables 3 and 4).

Generally speaking, the SCAD penalty has an edge over LASSO in selection features, namely those where the selective features are more consistent with their actual effects. The LASSO penalty may introduce more false interactions than the SCAD in the sparse high-dimensional models. Thus, _S_LASSO loses more true positives than _S_SCAD when controlling *FDR* estimation of stability selection at the desired level.

Overall, since the underlying interaction model is generally unknown, and a wide range of interaction models without marginal effects do exist [19], _S_SCAD is a valuable tool for discovering interactions without main effects and complement _S_LASSO in GWAS.

### Result of application

Two SNP-SNP interactions were significant in both the discovery and replication phases by _S_LASSO (Table 5), and four SNP-SNP interactions were significant in both phases of _S_SCAD selection. _S_SCAD contained all significant interactions identified by _S_LASSO. When using _S_SCAD, the two pairs (rs7839119-rs4524871 and rs2736100-rs40318) were shown to have significant interactions in both the discovery and replication populations. In contrast, neither of two pairs was validated as significant in the replication phase with _S_LASSO.

**Table 5:** **Empirical selection probability of significant SNP pairs by **_**S**_**LASSO and **_**S**_**SCAD under subsampling**

	**Trait**			**Nanjing Study**			**Beijing Study**			**Pooled Study**		
**SNP1(rs)**	**Gene1**	**SNP2(rs)**	**Gene2**	^***a***^ ***Π***		^**b**^**p**	***Π***		**p**	***Π***		**p**
				_**S**_**LASSO**	_**S**_**SCAD**	**Marginal**	_**S**_**LASSO**	_**S**_**SCAD**	**Marginal**	_**S**_**LASSO**	_**S**_**SCAD**	**Marginal**
rs7839119	PTK2	rs12544802	PTK2	0.724^c^*	0.702*	1.04 × 10^-6^	0.628*	0.940*	6.34 × 10^-2^	0.668*	0.801*	3.10 × 10^-4^
rs3781626	RAPSN	rs6018348	SRC	0.734*	0.680*	3.25 × 10^-3^	0.514*	0.654*	2.88 × 10^-3^	0.617*	0.824*	1.40 × 10^-4^
rs7839119	PTK2	rs4524871	MUSK	0.746*	0.734*	7.19 × 10^-4^	0.478	0.518*	4.98 × 10^-2^	0.600*	0.980*	5.02 × 10^-4^
rs2736100	TERT	rs40318	PIK3R1	0.586	0.830*	9.87 × 10^-6^	0.390	0.582*	2.22 × 10^-2^	0.696*	0.977*	2.51 × 10^-6^

^*a*^*Π* represents the empirical selection probability of SNP pairs under subsampling.

^b^p stands for the trend test p-value for simple marginal logistic regression.

^c^The significant SNP pairs under stability selection are coded by (*) to indicate its selection probability being higher than the threshold value (implied by *FDR* < 0.1).

For the _S_LASSO procedure, there were ten significant (*π*_*l*_ ≥ *π*_*thr*1_, *FDR* < 0.1) two-way SNP-SNP interactions in the Nanjing discovery study (Table 6). Among these ten SNP pairs, two were selected (*π*_*l*_ ≥ *π*_*thr*2_, *FDR* < 0.1) in the replication phase (Table 7). Both SNP-SNP interactions (rs7839119-rs12544802 and rs3781626-rs6018348) were verified in the replication phase. In an overall analysis that included discovery and replication datasets (5,408 subjects; 2,331 cases and 3,077 controls), the empirical selection probabilities of rs7839119-rs12544802 and rs3781626-rs6018348 interactions were 0.668 and 0.617, respectively; thus, indicating little statistical significance (*π*_*l*_ ≥ *π*_*thr*_, *FDR* < 0.1).

**Table 6:** **Empirical selection probability of significant SNP pairs in Nanjing study by the **_**S**_**LASSO**

**SNP1(rs)**	**Gene1**	**SNP2(rs)**	**Gene2**	^**a**^ ***Π***
				**Nanjing study **_**S**_**LASSO**
rs929087	*FASLG*	rs12544802	*PTK2*	0.964
rs4946933	*FOXO3*	rs11231740	*BAD*	0.890
rs2853462	*CHRNG*	rs7856889	*MUSK*	0.880
rs7445640	*TERT*	rs10733579	*MUSK*	0.824
rs411751	*PIK3R1*	rs939269	*PTK2B*	0.794
rs7839119	*PTK2*	rs4524871	*MUSK*	0.746
rs3781626	*RAPSN*	rs6018348	*SRC*	0.734
rs7839119	*PTK2*	rs12544802	*PTK2*	0.724
rs725787	*PTK2B*	rs5998196	*YWHAH*	0.688
rs6578141	*PTK2*	rs1940245	*MUSK*	0.636

^**a**^*Π* represents a predictor’s empirical probability of model inclusion under 500 times subsampling.

**Table 7:** **Empirical selection probability of significant SNP pairs in Beijing study by the **_**S**_**LASSO**

**SNP1(rs)**	**Gene1**	**SNP2(rs)**	**Gene2**	^**a**^ ***Π***
				**Beijing study **_**S**_**LASSO**
rs3779632	*PTK2B*	rs9644448	*PTK2*	1.000
rs2736100	*TERT*	rs11994882	*PTK2B*	0.904
rs10109684	*PTK2*	rs11231735	*BAD*	0.904
rs11994882	*PTK2B*	rs4983387	*AKT1*	0.840
rs6969923	*PIK3CG*	rs11997161	*PTK2*	0.788
rs2677764	*PIK3CA*	rs2821142	*MUSK*	0.784
rs4551415	*PTK2*	rs1359711	*MUSK*	0.740
rs1550099	*CHRNG*	rs10817088	*MUSK*	0.706
rs12466358	*CHRNG*	rs2565062	*PTK2B*	0.700
rs3791723	CHRNG	rs7839119	PTK2	0.684
rs7839119	*PTK2*	rs12544802	*PTK2*	0.628
rs9773817	*PTK2B*	rs6018088	*SRC*	0.624
rs479744	*FOXO3*	rs7952435	*BAD*	0.610
rs2736122	*TERT*	rs12945577	*CHRNB1*	0.598
rs10817082	*MUSK*	rs5994451	*YWHAH*	0.596
rs251398	*PIK3R1*	rs10733579	*MUSK*	0.530
rs3781626	*RAPSN*	rs6018348	*SRC*	0.514
rs4727666	*PIK3CG*	rs7856889	*MUSK*	0.506

^**a**^*Π* represents a predictor’s empirical probability of model inclusion under 500 times subsampling.

Under the _S_SCAD procedure, all four significant SNP-SNP interactions (rs7839119-rs12544802, rs3781626-rs6018348, rs7839119-rs4524871 and rs2736100-rs40318) were successfully replicated (Tables 5, 8, and 9). The empirical selection probabilities of rs7839119-rs12544802, rs3781626-rs6018348, rs7839119-rs4524871 and rs2736100-rs40318 interactions in the overall analysis, which included all 5,408 subjects, were 0.801, 0.824, 0.980 and 0.977, respectively. In turn, these results indicate statistical significance (*π*_*l*_ ≥ *π*_*thr*_, *FDR* < 0.1).

**Table 8:** **Empirical selection probability of significant SNP pairs in Nanjing study by the **_**S**_**SCAD**

**SNP1(rs)**	**Gene1**	**SNP2(rs)**	**Gene2**	^**a**^ ***Π***
				**Nanjing study **_**S**_**SCAD**
rs929087	*FASLG*	rs12544802	*PTK2*	0.904
rs2853462	*CHRNG*	rs7856889	*MUSK*	0.876
rs2736100	*TERT*	rs40318	*PIK3R1*	0.830
rs7445640	*TERT*	rs10733579	*MUSK*	0.786
rs411751	*PIK3R1*	rs939269	*PTK2B*	0.756
rs7839119	*PTK2*	rs4524871	*MUSK*	0.734
rs7839119	*PTK2*	rs12544802	*PTK2*	0.702
rs725787	*PTK2B*	rs5998196	*YWHAH*	0.692
rs3781626	*RAPSN*	rs6018348	*SRC*	0.680
rs4946933	*FOXO3*	rs11231740	*BAD*	0.640
rs6578141	*PTK2*	rs1940245	*MUSK*	0.604

^**a**^*Π* represents a predictor’s empirical probability of model inclusion under 500 times subsampling.

**Table 9:** **Empirical selection probability of significant SNP pairs in Beijing study by the **_**S**_**SCAD**

**SNP1(rs)**	**Gene1**	**SNP2(rs)**	**Gene2**	^**a**^ ***Π***
				**Beijing study**_**S**_**SCAD**
rs7839119	*PTK2*	rs12544802	*PTK2*	0.940
rs3779632	*PTK2B*	rs9644448	*PTK2*	0.828
rs10515077	*PIK3R1*	rs10817088	*MUSK*	0.658
rs3781626	*RAPSN*	rs6018348	*SRC*	0.654
rs2736122	*TERT*	rs12945577	*CHRNB1*	0.610
rs3639	*PTK2*	rs3781626	*RAPSN*	0.602
rs3791723	*CHRNG*	rs7839119	*PTK2*	0.594
rs2736100	*TERT*	rs40318	*PIK3R1*	0.582
rs9773817	*PTK2B*	rs6018088	*SRC*	0.574
rs3800230	*FOXO3*	rs7856889	*MUSK*	0.568
rs10980510	*MUSK*	rs3829603	*CHRNB1*	0.560
rs2677764	*PIK3CA*	rs2853668	*TERT*	0.556
rs411751	*PIK3R1*	rs9609396	*YWHAH*	0.526
rs9480867	*FOXO3*	rs11231741	*BAD*	0.522
rs7839119	*PTK2*	rs4524871	*MUSK*	0.518
rs4524871	*MUSK*	rs10980564	*MUSK*	0.502

^**a**^*Π* represents a predictor’s empirical probability of model inclusion under 500 times subsampling.

We also included the result of one permuted data set from the total 5,408 subjects combined. The selection probabilities of the _S_LASSO and _S_SCAD were 0.402 and 0.306, respectively. This corresponds to the maximum value of the test statistic for the permutation set. The cutoffs obtained from above inequality (6) for _S_LASSO and _S_SCAD with the significance (*FDR* < 0.1) were 0.593 and 0.560, respectively; thus, suggesting that the *FDR* calculated from the Meinshausen and Bülmann theorem is conservative. There appears to be little danger of selecting grossly inaccurate *FDR* when applying the Meinshausen and Bülmann theory.

## Discussion

Identifying interactions among multiple SNPs is both statistically and computationally challenging in large-scale association studies. The challenges include high-dimensional problems, computational capability, multiple testing problems, and genetic heterogeneity [20]. Many stochastic and heuristic detecting epistasis methods [21] could be used to analyze GWAS dataset. Wang *et al*. used AntEpiSeeker, a two-stage ant colony optimization algorithm (ACO), to identify epistasis [22]. Wan *et al.* proposed SNPRuler [23] based on both predictive rule inference, and two-stage design. Boolean operation-based screening and testing (BOOST) [24] involves only Boolean values, and allows the use of fast logic operations to obtain contingency tables. TEAM [25] exploits properties of test statistics to mitigate multiple testing problems. To date, there appears to be no one method free from model sensitivity.

In addition to non-parametric and model-free methods, many LASSO-based penalized parametric methods provide the estimation of parameter as the dimensionality increases, even if the number of variables is greater than the sample size. The coefficients of those none disease-associated SNPs will be zero in the penalized multivariate regression model. Thus, detecting interactions is equivalent with the variable selection problem under the framework of regression analysis. A broad range of simulations has demonstrated that the penalized regression method is a useful tool for detecting gene-gene interactions. However**,** the regularization choice in penalized regression is usually made by cross-validation that maximizes predictive accuracy in finite samples; although it does not necessarily induce the correct sparseness pattern for variable selection [26]. In our simulations, cross-validation often leads to the inclusion of too many noise variables, and induces instability of variable selection for the ordinary penalized regression method, such as LASSO or SCAD. A major hurdle for studying interactions in GWAS is the lack of efficient algorithms that can map different forms of interactions while keeping *FDR* under control [27]. _S_LASSO introduces stability selection into traditional LASSO. The stability selection procedure combines selection algorithms for high dimensional problems by sub-sampling. _S_LASSO dramatically reduces the number of false discovery rate, and could accurately identify crucial regions of GWAS; however, it is overly conservative, and may miss some important regions.

_S_SCAD procedure increases the power of detecting the interactions while controlling *FDR*. It attempts to provide more true interactions, but less noise terms than _S_LASSO. The above advantage could be attributed to the fact that running the LASSO-penalized procedure within stability selection results in more false positives than SCAD for each random sub-sample. Thus, the interactions causing noise as well as true interactions in the region both satisfy the threshold condition *π*_*thr*_ for selection. To control the number of falsely selected variables, the threshold must be very stringent. As a result, the _S_LASSO selection suffers a loss of power.

We analyzed a previously reported lung cancer dataset in Han Chinese, and confirmed significant interactions in the achPathway pathway, which supported the appropriate use of the proposed method. The observation of interactions between two closely located SNP pairs supports the hypothesis that some genetic variation in complex traits may hide in interactions between linked SNPs [28].

Application of the proposed procedure to GWAS data may ensure that the power of detection is reduced when over-stringent threshold *π*_*thr*_ conditions exist for the much increased ratio of SNPs to samples. A good alternative to derive genome-wide significant threshold is permutation. Unfortunately, genome-wide permutation in real GWAS of interactions is computationally prohibitive for the _S_SCAD selection. Partial search strategies based on biological knowledge [29] or the filtering of unimportant SNPs prior to analysis [30] could be adopted to reduce excessive computing burdens in early stage of genome-wide scale. These strategies are also necessary for the proposed method.

Under our current approach, high-dimensional data were primarily managed with sparse models. High correlations (individual SNPs that have a variance inflation factor (VIF) > 2 with other markers) were excluded. The chip data were pruned, and then analyzed with regression model method using a sparse constraint. Many common diseases may be associated with many SNPs with small to moderate effects. In this situation, we are considering group penalized methods in another paper.

## Conclusions

We developed a variable selection procedure (referred to as _S_SCAD selection). This procedure could control the *FDR* while maintaining the power to detect SNP-SNP interactions in association studies. In the pure interaction model, this procedure seems to overcome the conservativeness of _S_LASSO. The end result is that _S_SCAD, as a new technique in detecting interactions, can benefit the selection of _S_LASSO.

## Competing interests

The authors declare that they have no competing interests.

## Authors’ contributions

JG performed the simulations, participated in the data analysis, and prepared for the manuscript. YZ participated in the design of simulations, and helped to draft the manuscript. YW helped to perform the simulations. RZ, YQ and PZ participated in the data analysis. CW, TW,DY,WT, ZH, DL and HS acquired the data. FC conceptualized the study. All authors read and approved the final manuscript.
